# Symptoms, Etc., of the Œdema of the Glottis

**Published:** 1852-03

**Authors:** 


### *Symptoms, etc., of Œdema of the Glottis.*

In connection with the surgical treatment of Œdema of the Glottis, which is illustrated by the cases reported by Dr. Buck, contained in the foregoing article, we have thought some account of the symptoms, etc., of the affection may be acceptable to our readers. The following extracts are taken from an admirable essay on this subject embracing the history, diagnosis, and treatment of the disease, by Prof. Elisha Bartlett. The essay was published in the Western Journal of Med. and Surgery, May, 1850. We select the portions of the essay which possess most interest in a practical point of view. — EDITOR BUFFALO MEDICAL JOURNAL.

Œdematous laryngitis consists, in its essential anatomical element, in an infiltration and a consequent tumefaction of the lax submucous cellular tissue, upon the anterior surface of the epiglottis, and around the edges of the glottis. Bayle's original description of the diseased parts is in the following words: "In all cases, the edges of the glottis are swollen, thickened, white and trembling; they form a swelling more or less prominent, fully infiltrated with serum, which is pressed out from the cellular tissue with great difficulty, even after free incisions have been made in it. * * * The edges of the infiltrated and swollen glottis are disposed in such a manner, that every impulsion which comes from the pharynx turns them down upon the opening of the glottis, which is thus more or less obstructed, — while an opposite impulsion, from the trachea, throws these movable folds upward and outward, upon the sides of the opening of the glottis, rendering the orifice comparatively or entirely free. * * * The epiglottis is rarely intact; it is often very much swollen upon its edges." *

I have thought it best to add to the foregoing, even at the cost of some repetition, Hasse's minute and excellent description of the lesions. "The inflammation," he says, "assails the mucous membrane above the glottis, and particularly those of its folds which unite the epiglottis on the one hand with the root of the tongue and the arches of the palate, on the other, with the larynx, especially laterally, in the direction of the arytenoid cartilages — these folds being, as is well known, very lax and movable, and susceptible of great extension. * * * Inflammation of the epiglottis exhibits, after death, the whole of the mucous membrane between the root of the tongue and the glottis uniformly tumefied, so that the outlines of the epiglottis and arytenoid cartilages, together with the numerous folds and recesses in the vicinity of those parts have become effaced. This is the result of inflammatory effusion into the interspaces of the loose cellular texture, subjacent to the mucous membrane. In proportion to the intensity and duration of the inflammatory process, this exudation is sometimes of a purely serous and liquid nature, so as to flow away upon incision; sometimes blended with coagulable materials, and jelly-like; sometimes, again, mingled in various proportions with pus;

* Dic. des Sci. Med., vol. xviii. p. 510.

sometimes wholly purulent. Hence the tumor, which is always soft, lax, and tremulous, like jelly, varies greatly in color, being of a pale, or of a reddish yellow, sometimes of a dingy yellow or grayish white, and more or less opaque, — but, for the most part, superficially dotted with red. The swelling being dependent upon infiltration of the submucous cellular texture, cannot extend to the inferior surface of the epiglottis, because, there, no layer of cellular tissue exists; hence, the epiglottis has the aspect of having both its lateral edges bent over towards its nether surface, so as to form a narrow perpendicular groove, which is sometimes almost covered by the overhanging tumor. Neither does the swelling extend to the vocal ligaments — so that the term "*œdema glottidis*," is not strictly correct — but the tumor hanging down on each side, and in size often exceeding a pigeon's egg, overlays the glottis in such wise as to leave but a narrow opening towards the posterior part which allows the column of air to pass out during expiration, but is closed up by any attempt at inspiration. In no example recorded, did the œdematous infiltration beneath the mucous membrane show itself in any marked degree beyond the glottis." *

It will have been noticed in the foregoing descriptions, that while Bayle speaks only of a purely serous infiltration, Hasse states the infiltration to be sometimes serous, sometimes sero-purulent, and sometimes wholly purulent. There can be no doubt that the latter statement is the true one. Valleix says that, in more than three-quarters of the cases collected by himself, the infiltration was more or less mingled with pus. According to Valleix, in a few cases, the infiltration extends to the vocal cords.†

SEC. 3. *State of the Mucous Membrane.* — In a small number of cases, the infiltration above described, constitutes the only alteration. The mucous membrane, and the parts in the neighborhood of the glottis are quite free from any obvious disease. This, however, is not generally the case. In most instances, there are traces of inflammation in the mucous membrane, or some other lesion in or about the glottis. Valleix found the mucous membrane red in one-third of his cases, ulcerated in about one-sixth, in others detached from the subjacent tissues, and so on. * * * *

SEC. 1. *Mode of Access.* — Œdema of the glottis sometimes commences suddenly, with a formal and violent paroxysm of suffocative dyspnœa. This, however, is a rare occurrence. Valleix says that, of forty cases, the histories of which he had examined, two only commenced in this manner.‡ The access is generally somewhat gradual, although more or less rapid, — the disease usually reaching its stage of full development, in from a few hours to two or three days. The principal phenomena of this period are, a feeling of uneasiness in the larynx; an irresistible inclination to swallow, occasioned by the sensation of a foreign body in the pharynx, or upper part of the esophagus; efforts, by hawking, to clear the larynx and fauces; some difficulty of respiration; and slight hoarseness. The hand of the patient may be frequently carried to the seat of uneasiness in the larynx. ||

SEC. 2. *Pain in the Larynx.* — This pain, either spontaneous, or excited by pressure, is one of the earliest and also one of the most constant symptoms of the disease. It is mentioned as present, in some form or degree, in thirty-two of forty cases, by Valleix; and in only one of the remaining eight is its

---

* Hasse's Path. Anat., pp. 259 – 290. † Mem. de l'Acad. Roy. de Med., vol. xi., p. 107.

‡ Mem. de l'Acad. Roy. de Med., vol. xi., p. 1636. || Dic. des Sci., vol. xviii., p. 508.

absence formally stated. The pain is not often at all urgent, and in most cases does not attract the notice of the patient until his attention is called to it. In many instances, instead of positive pain, there is a pricking sensation in the larynx, some uneasiness on pressure, the feeling of a foreign body in the throat, or a sense of strangulation.*

Sec. 3. *Pain in the Pharynx.* — *Difficulty of Deglutition.* — In a certain proportion of cases there is pain in the pharynx, independent of the act of swallowing. Pain and difficulty of swallowing are present in a large majority of cases; in many instances they are extreme, and sometimes the act of swallowing becomes impossible. In three of Valleix's cases, drinks were returned by the nose. †

It results, then, from the foregoing statements, that pain about the throat, in the larynx or pharynx, or both, varying in kind and degree, spontaneous, or excited by pressure or motion, is an almost invariable accompaniment of the disease. It was noticed in thirty-eight of Valleix's forty cases.‡

Sec. 4. *Cough and Expectoration.* — Cough is neither an urgent nor a constant symptom. Bayle merely says that, in the progress of the disease there supervenes in some cases a slight and occasional cough. ‖ It is mentioned in only fourteen of Valleix's forty cases, and in the most of these it was slight. In some cases the cough is dry; in others there is more or less expectoration, varying in character in different patients, and in no way distinctive of the disease. Both the foregoing symptoms are of secondary importance.

Sec. 5. *Voice.* — One of the most constant symptoms of the disease consists in an alteration of the voice. This becomes rough and hoarse, then more and more muffled, and finally either nearly or altogether extinct. In only one of Valleix's cases had it the croupal character. §

Sec. 6. *Respiration.* — The capital and most characteristic symptom of œdema of the glottis, is the difficulty and embarrassment of the respiration. It is an invariable attendant upon the disease, and by the extreme and intolerable distress which it occasions, it concentrates and absorbs almost the whole attention both of the physician and the patient. It is necessary to describe this symptom in detail.

It has already been stated that, in a few cases, œdema of the glottis may commence with sudden and extreme dyspnœa. Generally, however, the difficulty of respiration, showing itself at a pretty early period of the disease, is at first only slight or moderate in degree; it subsequently goes on, more or less steadily and regularly, increasing in severity until it reaches its highest point of intensity.

There is a very striking peculiarity of the dyspnœa, first noticed by Bayle, and since by many observers. This peculiarity consists in the great difference in the difficulty of *expiration* and of *inspiration.* The latter is very much more difficult and laborious than the former. Bayle states, without any qualification, that while inspiration is very difficult, noisy, and hissing, expiration is always performed freely and easily. This statement requires some qualification. It is generally, but not absolutely, correct. In a large proportion of cases, the expiration does remain quite free and unembarrassed;

---

*Mem. de l'Acad. Roy. de Med. vol. xi, p. 136. †Mem. de l'Acad. Roy. de Med. vol. xi, 138.
‡ Mem. de l'Acad. Roy. de Med. vol. xi, p. 141. ‖ Dic. des Sci. Med. vol. xviii, p. 508.
§ Mem. de l'Acad. Roy. de Med. vol. xi, p. 146.

but not in all. Valleix says that in five of eighteen cases, the expiration was more or less difficult; but that in these cases the expiration was less difficult than the inspiration.

The difficult respiration is persistent and continued, but in most cases it is marked at intervals by paroxysms of extreme suffocative dyspnœa. The following is Bayle's vivid and truthful description of these frightful attacks: "When the paroxysm is violent," he says, "the patient, upright upon his seat, experiences an extreme difficulty in breathing; his shoulders are forcibly elevated, all his chest is in motion, inspiration is very laborious and noisy, while expiration remains free and facile; he is threatened with imminent suffocation; the face is pale and pinched, or flushed and swollen, with a fearful or wandering expression; some patients demand earnestly and imploringly that the larynx should be opened; others seek a cutting instrument with which to rid themselves of the substance that suffocates them; in many cases there are moments of fury, during which patients are tempted to put a violent end at once to their agony and life; they beat the bed with their hands, and in constant and violent agitation, and give utterance to cries of terror and despair. In these extreme paroxysms, and even in those that are much less violent, the pulse becomes unequal, irregular, and sometimes intermittent. When the fit has subsided, the breathing may become comparatively easy, but the pulse continues feeble and irregular. Not unfrequently, after a very short respite, another paroxysm or a series of paroxysms, carries off the patient; but more commonly, death takes place during the comparative calm that follows the fit." *

SEC. 7. *State of Pharynx.*—In most of the cases that have been reported, no mention is made of the appearance of the pharynx, and its examination seems to have been generally neglected. Valleix says, however, that of thirteen cases, in which the fauces were examined, there was only one which did not exhibit some appreciable lesion. In all the others there was manifest redness, more or less extensive, of the mucous membrane, with or without tumefaction; in two instances the semi-transparence of the swollen tissue showed that the tumefaction was owing to serous infiltration.† In four of the five cases reported by Dr. Buck, there was inflammation, more or less extensive, in and about the fauces.‡

SEC. 8. *Signs derived from Touch.* — The œdematous swelling of the lax sub-mucous cellular tissue, upon the upper side of the epiglottis, and along the edges of the glottis, constituting anatomically the disease under consideration, may be felt and recognized by the touch. This very important and positive means of diagnosis was first discovered by Tuilier; it was announced in a thesis presented to the Faculty of Medicine of Paris in 1815. Valleix and Dr. Buck, and there can be no higher authorities, add the testimony of their own repeated experience to the original statement of Tuilier. In Dr. Buck's five cases, before scarification was resorted to, the existence of the œdema of the epiglottis was positively ascertained by the touch. Dr. Buck speaks particularly of the swelling of the *epiglottis* only, but in the third case, he says, " while the end of the finger was pressing the epiglottis against the base of the tongue, the soft pulpy swollen edges of the glottis were felt rising up against it." ‖ Valleix says, that of twelve cases, in which the exploration

* Dic. des Sci. Med., vol. xviii, p. 509. † Mem. de l'Acad. Roy. de Med., vol. xi, p. 149.
‡ Trans. Amer. Med. Asso., vol. i, pp. 137, 144. ‖ Trans. Amer. Med. Asso., vol. i, p. 139.

by touch was methodically made, the œdematous swelling of the edges of the glottis was felt in eight. The swollen epiglottis, says Dr. Buck, conveys to the touch the sensation of a soft pulpy body, easily recognized and distinguished from the stiff rigid swelling of these parts in membranous laryngitis.

Sec. 9. *Physiognomy.* — The state of the face and its expression are such as might be looked for in a disease consisting essentially in a slow strangulation. The face is sometimes flushed, congested, and swollen; sometimes pale and sunken; the lips are livid, the eyes wild and staring; and the varying and commingled phases and degrees of bodily and mental agony, the dreadful sense of suffocation, and intense longing for fresh air, the consciousness of near and imminent danger, are vividly impressed upon the physiognomy.

Sec. 10. *General Symptoms.* — These are neither constant nor important. In some cases there is more or less febrile excitement; in others it is altogether wanting. The pulse is generally accelerated, becoming very rapid, feeble, and fluttering with the progress of the disease. The restlessness and agitation of the patient, at least before the gradual asphyxia is much advanced, are in proportion to the degree of dyspnœa. There is sometimes thirst; the appetite may be diminished or lost. If there is any disturbance of the stomach or bowels, it is quite accidental.

### CAUSES.

Sec. 1. *Preliminary.*— Œdematous laryngitis does sometimes occur spontaneously in persons at the time in good health, not suffering at the time with any general or local disease, and without any appreciable determining cause. This, however, is not common. It happened only three or four times in Valleix's forty cases. In most instances the disease is connected with some general morbid condition or with some local affection.

Sec. 2. *Local Disease in and about the Glottis.* — The frequency of this complication has already been stated in describing the anatomical lesions of the disease. These primary affections, acting as determining causes of the œdematous laryngitis, are thus enumerated by Valleix: 1. Simple inflammation of the laryngo-pharyngean mucous membrane. 2. Ulceration, acute or chronic, of the larynx, and sometimes of the pharynx. 3. Simple abscess of the pharynx, and sometimes of the larynx. 4. Alterations, more or less profound, of the laryngeal cartilages, with submucous abscesses, or disease of the mucous membrane. 5. In rare instances, inflammation of an organ more remote, such as the tongue.*

Sec. 3. *Convalescence from Fevers.* — The frequent occurrence of œdematous laryngitis during the convalescence from low fevers was noticed by Bayle, the first historian of the disease. As to the primitive form of this angina, he very truly says, it comes on most frequently during convalescence from febrile diseases of a grave character, such as adynamic or ataxic fevers.† There was an extraordinary frequency of the disease in the New York Hospital, between the months of December, 1847, and February, 1848. During this period, says Dr. Buck, the season was remarkably rainy and wet, accompanied with very little snow, and characterized by the prevalence of erysipelas and typhus fever, as well as an asthenic type in other diseases, both in and out of the hospital.‡

---

* Mem. de l'Acad. Roy. de Med., vol. xi, p. 120. † Dic. des Sci. Med., vol. xviii, p. 508.

‡ Trans. Amer. Med. Asso., vol. i, p. 135.

*Duration.* — The duration of the disease varies from only a few hours to two or three weeks. In a large majority of cases, however, unless the patients are relieved by a surgical operation, the disease is not prolonged beyond four or five days. This duration, according to Valleix, is generally longer in cases supervening upon chronic disease of the larynx, than in those that are primitive, or simply inflammatory.*

Death may take place during one of the suffocative paroxysms, or in the period of comparative calm that follows. The latter is more frequent than the former — a fact that was noticed by Bayle. Of the cases, constituting the basis of Valleix's memoir, three only terminated in the midst of a violent paroxysm; in seven, death took place during a period of comparative calm; but in all the others, constituting a considerable majority of the cases, the fatal termination took place, neither in the midst of a distinct paroxysm, nor during a period of well marked repose, but in a state of steadily progressive asphyxia, depending upon the persistent and gradually augmenting difficulty of respiration. In only one instance was death quite sudden.†

*Mortality and Prognosis.* — Œdematous laryngitis, unless relieved by a surgical operation, generally terminates in death. Bayle, when his first memoir upon the disease was written, in 1808, had seen seventeen cases — all of which were fatal. His subsequent experience did not induce him essentially to change his prognosis. Of the forty cases, collected by Valleix, nine terminated in recovery. This, it seems to me, is an unusually large proportion. It appears probable that the primitive form of the disease, occurring in persons otherwise in good health, is somewhat more susceptible of cure, than that which is connected with other lesions of the larynx, or supervenes in the course of some other disease.‡

*Mode of Operating by Dr. Buck.* — The patient being seated on a chair, with his head thrown back, and supported by an assistant, he is directed to keep his mouth as wide open as possible; and if there be any difficulty in this respect, a piece of wood an inch and a quarter in width, and half an inch in thickness, is to be placed edgewise between the molar teeth of the left side. The fore-finger of the left hand is then to be introduced at the right angle of the mouth, and passed down over the tongue till it encounters the epiglottis.

But little difficulty is generally experienced in carrying the end of the finger above and behind the epiglottis so as to overlap it and press it forward toward the base of the tongue. In some individuals the finger may be made to overlap the epiglottis to the extent of three-fourths of an inch.

Thus placed, the finger serves as a sure guide to the instrument to be used. The knife is then to be conducted with its concavity directed downward, along the finger till its point reaches the finger nail. By elevating the handle so as to depress the blade an inch to an inch and a half further, the cutting extremity is placed in the glottis between its edges; at this stage of the operation the knife is to be slightly rotated to one side and the other, giving it a cutting motion in the act of withdrawing it. This may be repeated without removing the finger two or three times on either side. The margin of the epiglottis, and the swelling between it and the base of the tongue may be scarified still more easily with the same instrument, or scissors curved

*Mem. de l'Acad. Roy. de Med. vol. xi, p. 162. †Mem. de l'Acad. Roy. de Med. vol. xi, p. 158.
‡ Mem. de l'Acad. Roy. de Med. vol. xi, p. 173.

flatwise may be employed for these parts, guided in the same manner as the knife.

Though a disagreeable sense of suffocation and choking is caused by the operation, the patient soon recovers from it, and submits to a repetition after a short interval. In every instance the operation has been performed twice, and in some three times.

Before proceeding to the operation, it has always been explained to the patient that the seat of his difficulty was a swelling at the top of the windpipe, preventing the air from entering, and the object of the operation was to cut it and let out fluid, and thus give him relief. This explanation corresponds so exactly with his own sensations, which refer to the top of the thyroid cartilage as the seat of obstruction, that he readily submits to the proposed operation, and renders all the co-operation in his power for its performance.

A slight hæmorrhage follows the scarifications, and should be encouraged by gargling with warm water. In one instance the quantity of blood mixed with sputa amounted to half a wineglassful.

